# Sjögren’s Syndrome and Silicosis – a Case Report

**DOI:** 10.3889/oamjms.2015.043

**Published:** 2015-04-23

**Authors:** Aleksandra Plavsic, Rada Miskovic, Jasna Bolpacic, Branka Šuštran, Aleksandra Peric-Popadic, Mirjana Bogic

**Affiliations:** 1 *Clinical Center of Serbia, Clinic for Allergology and Immunology, Belgrade, Serbia*; 2 *Serbian Institute for Occupational Health “Dr Dragomir Karajović”, Belgrade, Serbia*

**Keywords:** Sjögren’s syndrome, autoimmune diseases, silica, silicosis, environmental factor

## Abstract

Sjögren’s syndrome is an autoimmune disease of unknown etiology where immune response to self-antigens is believed to result from interactions between genetic and environmental factors. We describe the case of a patient who has been diagnosed with Sjögren’s syndrome based on typical clinical and immunological parameters. The clinical picture was dominated by the respiratory symptoms, and radiographic and multislice computed tomography examination of the chest showed certain changes characteristic of pneumoconiosis. Given that the patient has worked in a foundry where he has been exposed to the silica dust, he was subject to examination by occupational health specialists under the suspicion of lung silicosis, who confirmed the silicosis. This case report points to the possible connection between a professional exposure to silica and Sjögren’s syndrome. Occupational exposure to silica is a possible risk factor for the development of autoimmune diseases, and in the evaluation of patients with connective tissue diseases it is important to consider work-related history.

## Introduction

Sjögren’s syndrome is an autoimmune inflammatory disease characterized by the dysfunction of the exocrine glands and multiorgan involvement. As in the other autoimmune diseases, immunological response to self-antigens is complex and is assumed to occur in the interaction of genetic and environmental factors [1, 2]. The effect of chemical, physical and biological agents is important subject in the etiology of autoimmune diseases [3]. The exposure to the silica dust is associated with the development of respiratory diseases, namely to silicosis, a form of progressive pulmonary fibrosis [4]. However, the exposure to silica has been studied as a possible risk factor for the development of various autoimmune diseases, such as rheumatoid arthritis (RA), scleroderma, systemic lupus erythematosus (SLE), Sjögren’s syndrome and systemic vasculitis [5]. We are going to present the case of a patient diagnosed with Sjögren’s syndrome and silicosis.

## Case report

**Figure 1:**
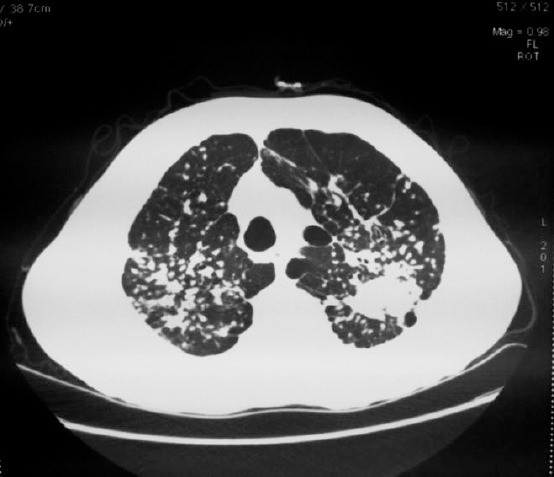
*Multislice computed tomography of the thorax*.

A male patient aged 53, a mould founder, was first treated at the Clinic for Allergology and Immunology, Clinical Centre of Serbia, in March 2014. Major complaints were shortness of breath, cough, and increased fatigue. These problems were present two years back in association with occasional peaks of fever, but he didn’t see a doctor until November 2013. Then, on chest X-ray a bilaterally and diffusely expressed interstitial pulmonary pattern with patchy and irregular intense shadows were observed, so he was referred to pulmonologist. During the treatment at the Clinic for Pulmology, Clinical Centre of Serbia, in December 2013, spirometrically a restrictive ventilation defect was registered with normal bronchoscopic test results (no signs of neoplastic process, test result of aspirates on acido-resistant bacilli negative). Multislice computed tomography (MSCT) of the chest showed numerous micronodular and macronodular changes and calcifications, partly flown together bilaterally in the lungs, with irregular changes in the left lobe which were more compatible with thick fibrous changes than with consolidations; smaller lymph glands up to 10mm in diameter in mediastinum, subcarinally calcified lymph glands up to 15mm in diameter (Figure 1). Immunoserological analyses showed a positive ANA-IIF using HEp-2 (titer 1:640), positive anti- SSB antibodies and positive anti -SSA antibodies. For this reason, the patient was sent to the Clinic for Allergology and Immunology, Clinical Centre of Serbia, with a suspected diagnosis of systemic connective tissue disease. In personal medical history data indicated that he has been treated for arterial hypertension, that he is a former smoker and consumes alcohol on a daily basis.

**Figure 2:**
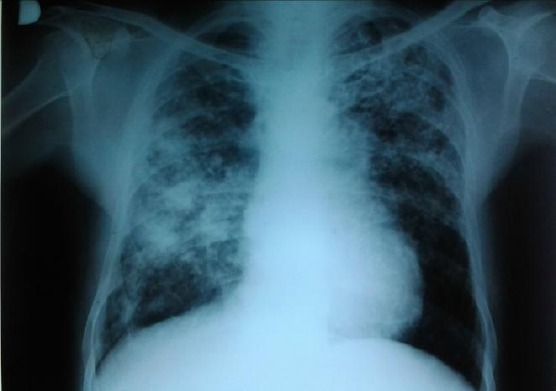
*Chest X-ray (the first hospitalization)*.

During the first hospitalization chest auscultation revealed diffuse reduced respiratory sound, elongation of expirium, an audible high-toned wheezing in the upper lung field, and delayed inspirium crackles at the bases of both lungs. Other physical findings were normal. Laboratory analysis registered increased values of the erythrocyte sedimentation rate, C-reactive protein, aspartate aminotransferase and ferritin; slightly lowered hemoglobin and total iron binding capacity; serum iron at the lower limit of reference values. The result of 24 h proteinuria was within the reference values. Chest X-ray showed micronodular and stained shadows billateraly, partly confluent (Figure 2). The abdominal ultrasound showed enlarged liver with 160 mm in the right lobe diameter, spleen with 155 mm in craniocaudal diameter, without focal changes. The ultrasound result did not show any peripheral lymphadenopathy. The signs of restrictive respiratory disorder were registered spirometrically. Ophtalmo-logical examination diagnosed keratoconjuctivitis sicca (the tear break-up time, Schirmer’s test and slit lamp examination after rose Bengal staining were conducted), whereas the dynamic salivary glands scintigraphy showed a reduced accumulative and excretory ability of parotid and submandibular glands. The patient was diagnosed with Sjögren’s syndrome on the basis of the following: subjective feeling of dry mouth and eyes lasting longer than three months, keratoconjuctivitis sicca, scintigraphic findings of reduced accumulative and excretory ability of parotid and submandibular glands, positive results of anti- SSA and anti-SSB antibodies. The glucocorticoids therapy was initiated (prednisolone per os at a dose of 30 mg a day, and then 20 mg a day), with antimalarial.

During the second hospitalization after 4 months, patient presented with a cough, exhaustion, increased fatigue, dryness in the mouth. There was a reduced respiratory sound in the lungs, with elongation of expirium, rare inspirium crackles, on both basal sides. Other findings were normal. Laboratory analysis showed increased values of the erythrocyte sedimentation rate and ferritin, other analyses were normal. A tuberculin skin test was negative; there were no acido-resistant bacilli in the sputum samples tests.

**Figure 3:**
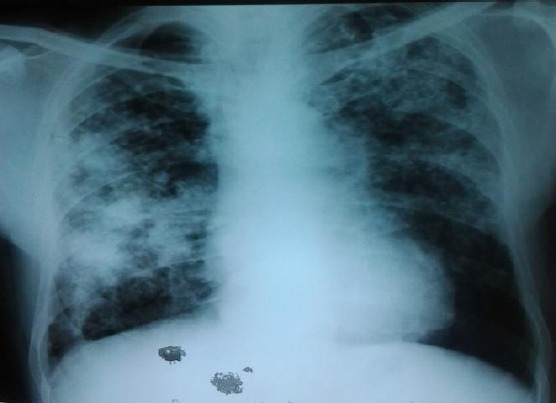
*Chest X-ray (the second hospitalization)*.

The virological analysis (HbsAg, anti HCV, anti HIV) were negative. Spirometrical signs of restrictive respiratory disorder were present, the lung diffusing capacity for carbon monoxide was decreased. A repeated chest X-ray showed both-sided micronodular, partly confluent shadows in the lower to middle lung field, flown together into larger stained shadows to the right (Figure 3). The ultrasound of the heart showed small tricuspid regurgitation 1+, and indirectly estimated systolic pressure in the right chamber was 45 mmHg. The liver and spleen were of normal characteristics and sizes on the ultrasound examination. As the patient has worked as a mould founder thus being exposed to the silica dust, we suspected a lung silicosis. The occupational health specialist was consulted, who suggested further examinations, which were conducted in October 2014. The lung silicosis of advanced stages was diagnosed. From the job description, it was concluded that the patient was exposed to high concentrations of free silica and the diagnosis of occupational diseases was made.

## Discussion

Sjögren’s syndrome is a chronic, autoimmune inflammatory disease which primarily affects exocrine glands and is characterized by sicca syndrome and systemic manifestation. It is assumed that both genetic and environmental factors have the role in the development of this disease [6, 7]. Although, the identification of genetic factors is very important, the role of various environmental factors in the pathogenesis of autoimmune diseases has been extensively studied, including the possible role of professional exposure to silica.

In the early 1950s it was noticed that there was a connection between silicosis and rheumatoid arthritis, as well as to silicosis and scleroderma [8, 9]. Since then, the connection between exposure to silica and/or silicosis and autoimmune diseases has occupied the attention of scientific community through case presentations, epidemiological and experimental models studies. However, the exact nature of this relationship is not still fully understood. The largest number of papers in literature refers to the relationship between exposure to silica and scleroderma and RA, but an association with SLE, as well as with systemic vasculitis has also been noticed.

In 1993 a group of Spanish authors conducted a prospective study on 50 workers from the factory for washing powder production with high level of silica [10]. The results showed that 32 of them had clinical characteristics of the systemic connective tissue disease: six with Sjögren’s syndrome, five with criteria for systemic sclerosis, three with SLE, five with overlap syndrome, and 13 were with insufficiently defined systemic disease of connective tissue. Antinuclear antibodies were discovered in 72% of the tested patients. The presence of antinuclear antibodies but also rheumatoid factor in silicosis patients was determined by other authors too [11, 12]. Rosenman and associates analyzed 463 persons with the diagnosis of silicosis and found a diagnose of RA in 24 of them, one was with scleroderma and one with SLE, that is, in those with silicosis they established a prevalence of 5.2% for RA, 0.2% for scleroderma and 0.2% for SLE [13]. A group of American authors has analyzed exposure to silica crystals in 265 patients with SLE, and comparing them to the control group, established the history of exposure to middle and higher levels of silica in 19% of the patients with SLE who worked on farms or in dusty trades, and 8% in control group [14]. The authors have concluded that in some individuals the exposure to silica crystals may promote the development of SLE. The American Thoracic Society considers that the exposure to silica crystals as a possible cause of SLE can be viewed only in patients with acute and progressive silicosis [15]. Slimani and associates have retrospectively analysed the medical records of the patients with connective tissue disease and a history of occupational exposure to silica [16]. They found four patients with scleroderma, three had RA, one SLE and one patient had Sjögren’s syndrome. The connection between ANCA-associated systemic vasculitis and exposure to silica is mostly based on case reports. After conducting meta-analysis of the current literature, a group of authors concluded that there is an association between silica exposure and risk for developing ANCA-associated vasculitis [17].

The literature data suggests a connection between exposure to silica and autoimmune diseases, as well as connection between silicosis and autoimmune diseases. However, the exact mechanism of autoimmune diseases development in persons exposed to silica is not entirely known. Silicon-dioxide is the most abundant mineral in earth’s crust and appears in amorphous and crystal forms. In many dusty trades, such as mining, sandblasting, granite cutting, tilling, rock drilling, brick laying, there is an exposure to respiratory particles of crystalline silica. After inhalation, dust particles of silica reach alveolar macrophages, where they are phagocyted, but because of their extreme toxicity macrophages themselves can be destroyed, and activate chronic inflammation through immune stimulation [5, 18]. A group of American authors has suggested a model in which inhalation of silica dust particles leads to activation and apoptosis of alveolar macrophages with possible consequent liberation of antigens, that is further ingested by activated macrophages or dendritic cells that are able to migrate to local lymph nodes [19]. In the lymph nodes macrophages and dendritic cells activate B and T lymphocytes leading to the autoimmune response. In the review of their experimental studies of patients with silicosis, Lee and associates have found the presence of different autoantibodies, an alteration of the CD95/Fas molecules, as well as the chronic activation of regulatory and responder T lymphocytes after the exposure to silica [20].

Recently, a role of adjuvants in the development of autoimmune diseases has been considered, and the term Autoimmune/inflammatory syndrome induced by adjuvants (ASIA) was introduced [21]. Adjuvant is defined as “any substance that acts to accelerate, prolong, or enhance antigen specific immune response” [22]. This syndrome is characterized by symptoms of fatigue, myalgia, arthralgia, neurological manifestations, fever, dry mouth, and is assumed to be caused by adjuvants, such as silicone, tetramethylpentadecane, pristane, aluminum [23]. Taking into consideration similar clinical characteristics of Sjögren’s syndrome and ASIA, their possible connection is also being viewed [24]. It was hypothesized that adjuvants can cause abnormal immune response directed to the epithelium of salivary and lacrimal glands and consequently tissue damage. In that context, an exposure to silica has been considered as a potential adjuvant in pathogenesis of Sjögren’s syndrome [24].

In the literature, the cases of associated exposure to silica and/or silicosis and Sjögren’s syndrome are rarely described. In the study of Sanchez-Romano and associates Sjögren’s syndrome was diagnosed in 6 out of 50 workers, in the study of Puisieux and associates there were 3 cases of primary Sjögren’s syndrome, one patient with Sjögren’s syndrome in the study of Slimani and associates, whereas Orriolos and associates have described a case of sicca syndrome and silicoproteinosis in a dental technician [10, 16, 25, 26]. A possible reason for this is that, unlike other systemic connective tissue diseases, especially SLE, scleroderma and ANCA- associated vasculitis, Sjogren’s syndrome is characterized by more organ-specific involvement. The clinical characteristics are mostly connected to the dysfunction of exocrine glands, and are less related to the lesions of other organic systems. Perhaps that is why sicca syndrome, joint pain, weakness and exhaustion are not considered as a part of a possible Sjögren’s syndrome, but as nonspecific symptoms. It should also be taken into account that most of the workers in mines and in dusty trades are male, so they are less suspected of possibly having a Sjögren’s syndrome, as it is more common in female persons.

The exposure to silica contributes to the development of autoimmune diseases, such as RA, scleroderma, SLE and ANCA-associated vasculitis [3, 27]. It is not yet clear whether the exposure to silica or the silicosis itself predispose the autoimmune disease development in some persons, or an autoimmune disease is the one that contributes to silicosis in persons exposed to silica [5]. It is possible that in some individuals with genetic predisposition, exposure to the silica is an additional risk factor, the trigger, which leads to autoimmune response. In any case, the data about professional exposure to silica should be carefully considered in the evaluation of patients with autoimmune diseases, and vice versa, in silicosis patients a possibility for different systemic autoimmune diseases should be taken into consideration, including Sjogren’s syndrome too.
